# Resin Cement/Enamel Interface: A Morphological Evaluation of the Acid-Base Resistant Zone, Enamel Etching Pattern, and Effect of Thermocycling on the Microshear Bond Strength

**DOI:** 10.3290/j.jad.b3974603

**Published:** 2023-03-20

**Authors:** Rose Y. Kumagai, Tomohiro Takagaki, Takaaki Sato, Toru Nikaido, Marcelo Giannini, Andre Reis, Junji Tagami

**Affiliations:** a PhD Candidate, Cariology and Operative Dentistry, Graduate School of Medical and Dental Sciences, Tokyo Medical and Dental University, Tokyo, Japan; Dental Research and Graduate Studies Division, Department of Restorative Dentistry, Guarulhos University, Guarulhos, Brazil. Performed bond strength test, SEM analysis, thermocycling, wrote the manuscript.; b Associate Professor, Department of Operative Dentistry, Division of Oral Functional Science and Rehabilitation, Asahi University, School of Dentistry, Mizuho, Japan. Idea, study design, wrote the manuscript.; c Assistant Professor, Cariology and Operative Dentistry, Graduate School of Medical and Dental Sciences, Tokyo Medical and Dental University, Tokyo, Japan. Idea, study design, wrote the manuscript.; d Professor and Chairman, Department of Operative Dentistry, Division of Oral Functional Science and Rehabilitation, Asahi University, School of Dentistry, Mizuho, Japan. Idea, study design, wrote the manuscript.; e Clinical Associate Professor, Department of Restorative Dental Sciences, Division of Operative Dentistry, University of Florida College of Dentistry, Gainesville, FL, USA. Idea, study design, statistical analysis, wrote the manuscript.; f Associate Professor, Department of Restorative Dental Sciences, Division of Operative Dentistry, University of Florida College of Dentistry, Gainesville, FL, USA. Idea, study design, wrote the manuscript.; g Professor and Chairman, Cariology and Operative Dentistry, Graduate School of Medical and Dental Sciences, Tokyo Medical and Dental University, Tokyo, Japan. Idea, study design, wrote the manuscript.

**Keywords:** dental bonding, dental cements, resin cements.

## Abstract

**Purpose:**

To evaluate the effects of etching mode (self-etch and etch-and-rinse) on acid-base resistant zone (ABRZ) formation at the resin cement/enamel interface and enamel etching pattern, as well as the effects of thermocycling (0, 5000, and 10,000 cycles) on the enamel microshear bond strength (μSBS) mediated by dual-cure resin cements (DCRC).

**Materials and Methods:**

Two DCRC were used in 4 groups: Panavia V5 in self-etch (V5NE) and etch-and-rinse mode (V5E); and Estecem II in self-etch (ENE) and etch-and-rinse mode (EE). For ABRZ observation, the bonded interface was subjected to a demineralizing solution. The morphological attributes of the interface and etching patterns were observed using FE-SEM. For μ-SBS, cylinders with a 0.79-mm internal diameter and 0.5-mm height were made with DCRC and tested in shear after 0, 5000, and 10,000 thermal cycles (TC) (5°C and 55°C) (n = 10).

**Results:**

The formation of an enamel ABRZ was observed in all groups with different morphological features between self-etch and etch-and-rinse groups. A funnel-shaped erosion beneath the interface was present using V5NE and ENE modes where enamel was dissolved, while ABRZ formation was confirmed and no funnel-shaped erosion was noticed using V5E and EE. No significant differences in μSBS were observed between resin cements. However, significantly lower µSBSs were recorded when the self-etching mode was used. Thermocycling resulted in a significant reduction in µSBS for all groups.

**Conclusion:**

Selective enamel etching should be recommended to improve the interfacial quality when dual-cure resin luting cements are used.

Indirect esthetic restorations are widely used, for which resin cements are the luting materials of choice because of their advantageous characteristics, such as high bond strength, wear resistance, low solubility, esthetic shades, etc.^2,7,21,26,36^ Furthermore, the ability to bond restorations to tooth structure reinforces both the dental restoration and the dental tissue,^4,6^ and reduces microleakage, marginal staining, postoperative sensitivity, and recurrent caries.^12^ According to the adhesive approach for cementation, resin cements can be divided into three groups: etch-and-rinse systems in which phosphoric acid etchant is performed prior to adhesive application; self-etching systems, in which self-etching primers or self-etching adhesives are employed; and self-adhesive cements, when no adhesive is used.^30,39^

An acid-base resistant zone (ABRZ) adjacent to the dentin hybrid layer in self-etching adhesives was first observed in 2004 by Tsuchiya et al.^37^ This zone seals the restoration margins, which helps prevent secondary caries and increases the durability of the restoration.^22-25^ Li et al^15^ described a similar ABRZ in enamel using a two-step self-etching primer system.^15^ However, there is no information about formation of ABRZ on enamel using dual-curing resin cements.

A multistep application technique for luting cements can be a time-consuming, sensitive technique that may compromise bonding effectiveness.^16^ Thus, some manufacturers no longer recommend a separate etching step for dentin and enamel. However, it is not clear whether omitting phosphoric-acid etching of enamel is a safe choice.

Therefore, as there is no previous paper describing ABRZ formation at the resin-cement/enamel interface, although this class of material is used ever more frequently in clinical routine, the aims of this study were: to evaluate the microshear bond strength (μSBS) of two dual-curing self-etching resin cements applied with or without phosphoric-acid conditioning of enamel after 0, 5000, and 10,000 thermal cycles; illustrate the ultramorphology of the resin-cement/enamel interface after acid-base challenge (formation of ABRZ); and determine the enamel-etching pattern of self-etching primer-coated surfaces with or without phosphoric-acid pre-etching. The null hypothesis to be tested was that acid etching prior to resin cement application does not influence the μSBS of two dual-curing self-etching resin cements applied with or without phosphoric-acid conditioning on enamel after 0, 5000, and 10,000 thermal cycles.

## Materials and Methods

Sixty-six noncarious human molars were used in this study to test two different dual-curing resin cements. Sixty teeth were used for the microshear bond strength test, four for FE-SEM observation after acid-base challenge, and two for FE-SEM observation of the primer-coated enamel-surface etching patterns with or without prior phosphoric-acid etching. The teeth were collected after the donors’ informed consent was obtained according to the protocol approved by the Research Ethics Committee (# 641.271). The teeth were stored at 4°C in saline solution with 0.01% thymol and used within 6 months after extraction.

From each tooth, the root was removed and the crown was sectioned in the mesio-distal direction using a low-speed diamond saw (Isomet, Buehler; Lake Bluff, IL, USA). Afterwards, the buccal and lingual enamel surfaces obtained were embedded in epoxy resin (EpoxiCure, Buehler) with the enamel surfaces facing outwards. These were then wet ground with 600-grit SiC paper for 1 min in order to expose flat enamel surfaces, resulting in 120 enamel specimens.

### Microshear Bond Strength (μSBS)

The primers used in combination with each dual-curing resin cement were applied according to the respective manufacturer’s instructions (Table 1), except for the variation consisting of 35% phosphoric acid for 10 s in the etch-and-rinse groups (K-etchant Syringe, Kuraray Noritake; Tokyo, Japan). The enamel etching time was determined according the phosphoric acid manufacturer’s instructions. Following the enamel-specimen preparation described above, the specimens were randomly divided into 4 groups: V5NE (Panavia V5, shade Universal; Kuraray Noritake) without pre-etching; V5E Panavia V5 with pre-etching; ENE (Estecem II, shade Universal, Tokuyama Dental; Tokyo, Japan) without pre-etching; and EE (Estecem II) with pre-etching.

**Table 1:** Manufacturers, compositions, instructions for use, and batch numbers of the dual-cure resin cements and respective primers

Material (Manufacturer) Lot No.	Composition	Procedure
Panavia V5 (Kuraray Noritake) SU0035 / 3f0051 Shade Universal	Tooth primer (pH 2.0): 10-MDP, original multifunctional monomer, new polymerization accelerator, HEMA, water, stabilizer. Cement: bis-GMA, TEG-DEMA, aromatic multifunctional monomer, aliphatic multifunctional monomer, new chemical polymerization accelerator, dl-camphor quinone, photopolymerization accelerator, surface treated barium glass, fluoroalumino-silicate glass, fine particulate filler	Apply and leave primer for 20 s, gently air dry, apply paste from auto-mix syringe, light cure for 20 s
Estecem II (Tokuyama Dental) 001057 / A009B1 Shade Universal	Bondmer Lightless (pH 2.2) Bond A: phosphoric acid monomer (3D-SR monomer), HEMA, bis-GMA, TEG-DMA, acetone, MTU-6, others Bond B: borate, peroxide, acetone, isopropyl alcohol, water, silane coupling agent, others Cement: paste A: bis-GMA, TEG-DMA, bis-MPEPP, silica-zirconia filler (74% weight / 61% volume). Paste B: bis-GMA, TEG-DMA, bis-MPEPP, silica-zirconia filler (74% wt/ 61% vol), camphorquinone, peroxide	Dispense one drop of Bond A and Bond B into the dispensing well and mix (complete the application within 1 min after dispensing). Apply on the surface and wait 10 s, gently air dry until it becomes motionless. Then apply strong air stream to the surface, apply paste from auto-mix syringe, light cure at least 20 s
K-etchant Syringe (Kuraray Noritake) 1L0033	35% phosphoric acid aqueous solution and colloidal silica	Apply and leave for 10 s, rinse thoroughly and dry

10-MDP: 10-methacryloyloxydecyl dihydrogen phosphate; HEMA: 2-hydroxyethyl methacrylate; MTU-6:6-methacryloyloxyhexyl 2-thiouracil-5-carboxylate; bis-GMA: bisphenol-glycidyl methacrylate; bis-MPEPP: bisphenol A polyethoxy methacrylate; TEG-DMA: triethylene glycol dimethacrylate.

After self-etching primer application, three cylindrical translucent molds made of Tygon tubing (TYG-030, Saint-Gobain Performance Plastic; Solon, OH, USA) with an internal diameter of 0.79 mm and a height of 0.5 mm^14,15^ were positioned onto exposed, flat enamel surfaces. Then, the dual-curing resin cement was carefully inserted into each mold and light cured for 20 s using an LED light-curing unit (VALO, Ultradent; South Jordan, UT, USA) at 1400 mW/cm^2^. The translucent molds were removed with a thin steel cutting blade after 24 h of storage in water at 37°C. Enamel samples were subdivided into 3 subgroups according to the thermocycling regimen (n = 10): 0, 5000, and 10,000 thermocycles. Specimens were subjected to two water baths of 5°C and 55°C with a dwell-time of 30 s at each temperature (Thermocycling K178, Tokyo Giken; Tokyo, Japan) prior to the μSBS test.

Subsequently, each specimen was attached to the testing device with cyanoacrylate glue and placed in a universal testing machine (EZ-test-500N, Shimadzu; Kyoto, Japan) to determine the μSBS. A thin wire (diameter 0.20 mm) was looped around the resin-cement cylinder, contacting it at the resin-cement interface. Load was applied at a crosshead speed of 1.0 mm/min until failure occurred. Data from the μSBS test were analyzed using three-way ANOVA (resin-cement vs etching mode vs thermocycling) and Tukey’s test at a pre-set confidence level of 0.05 (IBM SPSS version 20.0.0, IBM; Armonk, NY, USA).

Failure mode was analyzed using a confocal laser scanning microscope (CLSM; VK-X150/X160, Keyence; Osaka, Japan) at 240X magnification. The fractured interface was classified as one of three types: CE (cohesive failure in enamel), AD (adhesive failure), and CR (cohesive failure in resin-cement). Instead of classifying failures as mixed, the percent area of each failure type in each specimen was recorded.

### FE-SEM Observation after Acid-Base Challenge

Enamel specimens were prepared as described for the µSBS test. A 2-mm-thick layer of the dual-curing resin cement was applied and light cured for 20 s. Each specimen was stored in distilled water at 37°C for 24 h; afterwards they were halved perpendicular to the bonding interface, re-embedded in epoxy resin (Epoxicure Resin, Buehler; Lake Bluff, IL, USA), and left overnight. Subsequently, the specimens were ground with SiC papers from 600-grit to 1200-grit for 1 min and subjected to an acid-base challenge. For the acid challenge, each specimen was stored in buffered demineralizing solution (pH 4.5, 2.2 mmol/l CaCl_2_, 2.2 mmol/l NaH_2_PO_4_, and 50 mmol/l acetic acid) for 4.5 h. In the base challenge, the specimens were immersed in 5% NaOCl with ultrasonication twice for 10 min each time, and rinsed immediately afterwards with tap water for 30 s to remove any debris or enamel proteins on the demineralized structures. Then, a 4-META/MMA-TBB resin (Super Bond C&B, Sun Medical; Shiga, Japan) was applied without acid etching the treated surface to prevent wear or fracture of the remaining structure during cutting and polishing. The specimens were sectioned perpendicularly to the resin-cement/enamel interface and polished with SiC papers from 600-grit to 1200-grit, followed by polishing with diamond pastes (Struers; Copenhagen, Denmark) for 1 min down to a particle size of 0.25 μm with ultrasonication of 2 min between each particle size. To bring the resin-cement/enamel interface into sharp relief, argon-ion etching (EIS-200ER, Elionix; Tokyo, Japan) was performed for 30 s with an accelerating voltage of 1 kV and an ion-current density of 1.5 mA/cm^2^ on the polished surfaces. FE-SEM (S-4500, Hitachi; Tokyo, Japan) analysis was conducted with an accelerating voltage of 15 kV after platinum sputter-coating.

### FE-SEM Observation of Enamel Etching Pattern

For FE-SEM observation of the enamel-etching pattern of primer-coated surfaces with or without prior phosphoric-acid etching, enamel specimens were prepared from two human molars as described previously.

For etched and non-etched groups, after self-etching primer application, specimens were immersed in an ultrasonic bath with acetone for 3 min in order to dissolve the primer and dehydrate specimens. Then, specimens were sputter-coated with platinum and observed in FE-SEM with an accelerating voltage of 15 kV.

## Results

### Microshear Bond Strength (μSBS)

Mean (± SD) μSBSs in MPa are presented in Table 2. Three-way ANOVA revealed no significant differences for the factor “resin cement” (p = 0.8008). However, it detected significant differences for the factors “etching mode” (p < 0.0001), “thermocycling” (p = 0.0033), and the interaction among factors (p < 0.0001).

**Table 2:** Mean bond strengths (in MPa) for the two dual-cure resin cements applied with and without prior phosphoric-acid etching (self-etch and etch-and-rinse mode) in enamel after 0, 5000, and 10,000 thermocycles

Cement	Mode	TC 0	TC 5000	TC 10,000
Panavia V5	Self-etching	22.5 (4.6)^Ba^	15.2 (5.7)^Bb^	13.3 (4.6)^Bb^
	Etch-and-rinse	27.1 (4.7)^Aa^	20.6 (5.7)^Ab^	16.4 (3.6)^Ab^
Estecem II	Self-etching	20.9 (5.0)^Ba^	13.9 (3.5)^Bb^	11.1 (3.3)^Bb^
	Etch-and-rinse	28.3 (4.0)^Aa^	22.5 (3.8)^Aab^	18.9 (4.1)^Ab^

Means followed by different letters (superscript capital letters compare etching modes within the same thermocycling time and for the same resin cement, superscript lower-case letters compare thermocycling times for the same resin cement and same etching mode). No significant differences were observed between resin cements. Confidence level set at 0.05.

With regard to etching mode, for both cements at all evaluation periods, etching enamel with phosphoric acid resulted in significantly higher bond strengths (p < 0.0001). Thermocycling resulted in a significant reduction in bond strengths for both resin cements regardless of the etching mode (p = 0.0033). The only exception was observed for EE: There was no significant difference in µSBS for EE from 0 to 5000 thermocycles, but µSBS was significantly lower after 10,000 cycles.

**Fig 1:**
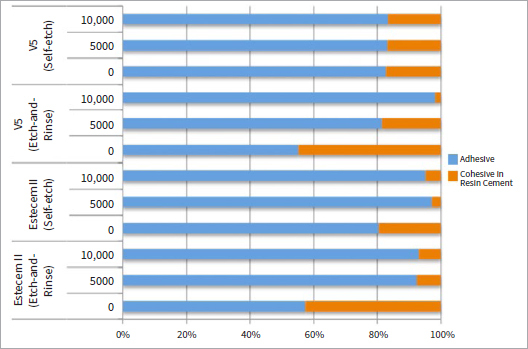
Failure mode distribution (%).

**Fig 2:**
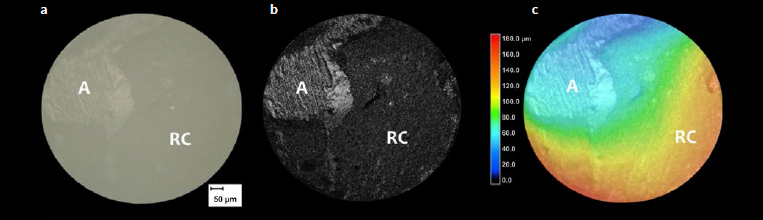
Representative CLSM images of the failure modes after μSBS testing (200X magnification). a: light-microscopic mode; b: CLSM mode; c: 3D topological analysis mode. A: adhesive failure; RC: cohesive failure within resin cement.

Descriptive data of failure mode analysis are shown in Fig 1. The predominant failure mode in all groups was adhesive. However, for both resin cements applied in etch-and-rinse mode without thermocycling, a higher percentage of cohesive failures in resin cement was observed. No cohesive failures in enamel were observed. Figure 2 shows a typical example of failure, as observed using CLSM.

### FE-SEM Observation after Acid-Base Challenge

**Fig 3:**
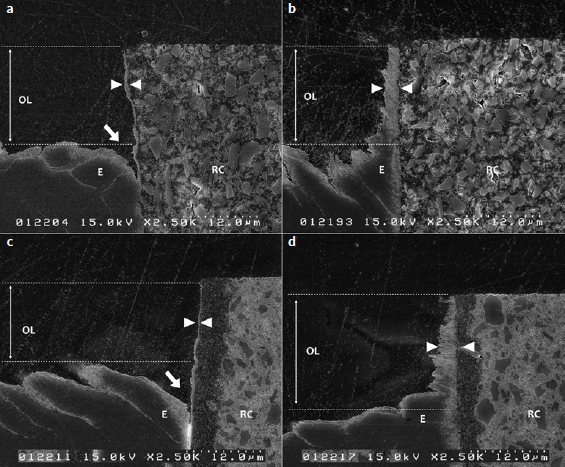
Representative FE-SEM images of the ultrastructure of enamel/resin-cement interfaces after acid-base challenge. a) V5NE; b) V5E; c) ENE; d) EE. E: enamel. Between white arrowheads: ABRZ.RC: resin cement; OL: outer lesion. White arrow in Figs 3a and 3c point to the funnel-shaped erosion at the interface. Acid-base challenge creates an outer lesion (OL) with a depth of approximately 12 to 17 μm. In all groups, an ABRZ (between arrowheads) was detected beneath the bonding layer that was approximately 0.4 µm thick in self-etching groups and 3 μm in etch-and-rinse groups. Original magnification 2500X.

The formation of an enamel ABRZ was observed in all groups, although with different morphological features. Figure 3 shows typical interface morphologies of each group after acid-base challenge. An outer lesion (OL) – approximately 12.0 to 17.1 μm deep – created by mineral loss due to the acid-base challenge was observed in all groups.

In contrast, V5NE and ENE specimens presented an ABRZ with approximately 0.6 μm and 0.4 μm thickness, respectively, and funnel-shaped erosion along the interface was detected beyond the OL, where enamel was dissolved and detached from the bonding layer. The width of the eroded area beneath the ABRZ was 11.4 μm in V5NE specimens and 14.6 μm in ENE specimens at the top of the eroded area.

In V5E and EE specimens, a bonding interface without gaps or defects was formed. An ABRZ approximately 2.4 μm thick was observed in the V5E group, and the EE group presented an ABRZ about 3 μm thick. Furthermore, no demineralization beyond the outer lesion (OL) and no funnel-shaped erosion were detected along the ABRZ in the phosphoric-acid pre-etching groups.

Slight morphological differences in ABRZ were observed between V5E and EE specimens. The ABRZ of V5E seemed more uniform from the outer surface towards the inside. In contrast, the ABRZ of EE appeared thinner closer to the outer surface, suggesting that the ABRZ of EE could be more susceptible to the acid-base challenge.

### FE-SEM Observation of Enamel Etching Pattern

**Fig 4:**
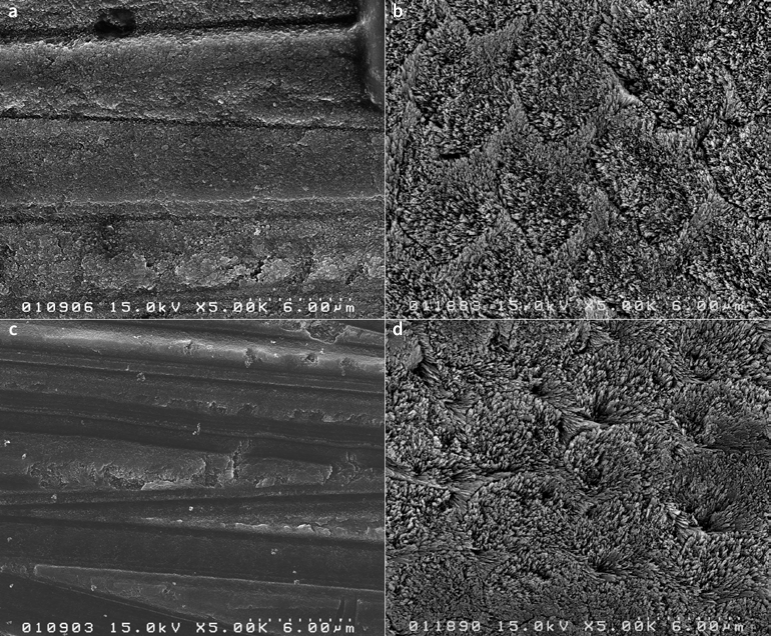
FE-SEM observation of enamel-surface etching pattern (5000X magnification). a) V5NE; b) V5E; c) ENE; d) EE. Figs 4b and 4d: surfaces etched with phosphoric acid and primed show microporosities, enamel crystals, and a honeycomb-like appearance. Figs 4a and 4c: without prior phosphoric-acid etching, both groups show the surface covered by a smear layer with no clear signs of demineralization.

Etching patterns of primed enamel surfaces with or without prior phosphoric-acid etching are shown in Fig 4. In groups V5E and EE, exposed crystallites within enamel prisms were observed. V5NE and ENE groups showed minimal or no signs of demineralization or exposed enamel prisms. Phosphoric-acid etched and primed groups exhibited a honeycomb pattern that is caused by preferential dissolution of the enamel prism cores; the prism peripheries were also observed.

## Discussion

In the present investigation, two dual-curing self-etching resin cements systems (Panavia V5 and Estecem II) were tested on human enamel using two different pre-treatments: with or without phosphoric-acid etching prior to application of the self-etching primers. The μSBS test was chosen as it enables bond testing in small areas^27^ and also because sectioning is not necessary to obtain specimens, which could otherwise produce microcracking in a brittle substrate such as enamel.^8,33,34^

The null hypothesis of this study, that phosphoric-acid etching prior to application of a self-etching resin cement would not influence the μSBS after 0, 5000, or 10,000 thermocycles, was rejected. Thus, etching enamel with 35% phosphoric acid positively influenced all parameters evaluated.

Except for previous etching with 35% phosphoric acid in groups V5E and EE, the materials were used according to manufacturers’ instructions. Compared to dentin, enamel presents a higher mineral content with a crystalline structure.^17^ Enamel contains approximately 96% hydroxyapatite by weight, and the remainder consists of water and organic material.^11,40^ Bonding to enamel is achieved by micromechanical interlocking via resin-monomer diffusion into the pre-treated enamel, followed by polymerization.^35^ No significant differences in μSBS were observed between the dual-curing resin cements tested, but significantly higher bond strengths were observed when phosphoric-acid pre-etching was used.

Thermocycling subjected the specimens to extreme temperature differences to simulate intraoral conditions, generating repetitive contraction and expansion stresses in the dental substrate and also in the restorative material. Because of these stresses, cracks can propagate along the interface, allowing fluid infiltration. The actual number of thermal cycles likely to be experienced in vivo is not precisely known, but a provisional estimate of approximately 10,000 thermocycles per year has been suggested.^9^ The μSBS of all groups tested in this study decreased after thermocycling. Panavia V5 and Estecem II in self-etch and etch-and-rise mode did not show significant differences between 5000 and 10,000 thermocycles. When applied in etch-and-rinse mode, Estecem II showed no significant differences between 0 and 5000 thermocycles, but a significant reduction was detected between 0 and 10,000 thermocycles. No significant differences were observed between resin cements. Compared with the well-established 10-MDP monomer present in Panavia V5, the relatively new functional monomer 3D-SR contained in Estecem II seems to perform similarly well immediately after application as well as after in-vitro thermocycling.

CLSM was chosen in this study to analyze failure mode. There is evidence^42^ that when the shear bond strength test is performed, stresses often concentrate in the substrate and failure cannot be considered at the interface itself. Using CLSM, it is possible to observe the thickness of the remnant resin cement at the fracture location, verifying whether the specimen and the µSBS test apparatus were correctly positioned at the moment of fracture. In addition, due to the limited enamel surface available on human teeth, the reduced dimensions of μSBS vs conventional SBS specimens are advantageous.^33^

The pH of most current self-etching adhesives is classified as “mild” (pH>2) or “ultra-mild” (pH>2.5).^1,3,5,19,20,28,41^ This pH range can be effective for dentin bonding, but it seems to be insufficient for enamel conditioning, which is even more critical when uncut enamel is involved.^10,13^ The pH of Panavia V5 Tooth Primer is 2.0 and the pH of Bondmer Lightless used with Estecem II is 2.2.^22^ Another point to consider is a direct correlation between the pH and compatibility of universal adhesives with self- and dual-curing resin cements.^10^ It has been found that more acidic adhesives present lower compatibility with these materials.^32^

The enamel-etching patterns found in this study were similar to those reported by Li et al^14^ and Sato et al.^31^ When the primer was applied without prior phosphoric-acid etching (Figs 1 and 2), the smear layer covered the entire surface, and minimal or no signs of etching or exposed enamel prisms were noted. This demonstrates that the self-etching primers were not acidic enough to etch the enamel surface. On the other hand, when prior phosphoric-acid etching was performed, it was possible to identify enamel crystallites and a honeycomb pattern (Figs 3 and 4).

Similar to all-in-one adhesives, the primers used with the dual-curing resin-cement in this study contain one or more functional monomers, which are important in etching enamel and/or dentin, in that they enhance monomer penetration and also participate in chemical interaction potential.^38^ Panavia V5 Tooth Primer contains 10-MDP, an acidic functional monomer that demineralizes the smear layer as well as the subjacent substrates. In Bondmer Lightless, the primer used with Estecem II cement, 3D-SR monomer is the phosphoric-acid monomer responsible for demineralization of dental hard tissue and enhanced adhesion via interaction with tooth calcium.

Tsuchiya et al^37^ reported the formation of the acid-base resistant zone in dentin. Li et al^15^ described the formation of a similar zone in enamel. They investigated the effects of 10-MDP and phenyl-P on the morphology of the adhesive-enamel interface after an acid-base challenge, finding that only in adhesives containing 10-MDP was the enamel ABRZ present.^15^ Nikaido et al^22^ reported the formation of an enamel/dentin acid-base resistant zone by three experimental adhesives containing different functional monomers: 10-MDP, 3D-SR, and 4-META, with pH values of 1.9, 2.0, and 2.2, respectively. These previous studies support our observations that an enamel ABRZ formed in all groups in which the functional monomers were 10-MDP and 3D-SR, ie, in Panavia V5 Tooth Primer and Bondmer Lightless, respectively.

In addition, when enamel was first etched with phosphoric acid (V5EE and EE), the ABRZ was quite apparent, thicker, and irregular. In contrast, when no prior phosphoric-acid etching was performed, the ABRZ was thin and regular, which can be explained by the mild etching potential of the primer. In a previous study,^14^ the same pattern of ABRZ formation was shown when phosphoric acid was adjunctively used with a mild self-etching adhesive.

The mechanism of enamel ABRZ formation remains unclear. However, it is supposed that its formation is favored by the penetration of functional monomers into the micropores created by enamel etching, the self-etching primer, and the chemical interaction between the functional monomer and hydroxyapatite.^15,17,18,22^

Although the formation of an ABRZ in enamel by primers or adhesives containing functional monomers has been reported,^31^ there is no previous study testing dual-curing resin cements. These are becoming increasingly popular among clinicians thanks to a fewer number of steps, which reduced the probability of error and saves chair time.

For this study, ground enamel surfaces were used, but intact enamel is also present in clinical situations. Intact enamel should be considered in future studies, taking minimally invasive preparations and esthetic interventions into account, when indirect restorations can be bonded even without preparation. In addition, the resin cements tested were dual-curing, so that it would be interesting to study these cements after chemical curing only.

## Conclusion

Acid etching of should be recommended to improve the interfacial quality of the adhesive interface when dual-curing, self-etching resin cements are used.

## Clinical relevance

Acid etching of should be recommended to improve the interfacial quality of the adhesive interface when dual-curing, self-etching resin cements are used.
